# Treatment of Inflamed Testicle by Puncture

**Published:** 1871-04

**Authors:** 


Treatment of Inflamed Testicle by Puncture.—At a late meeting of the Medical Society of Londun (*The Lancet*, Nov. 12, 1870), Mr. Henry Smith made some observations on the excellent results he had obtained from puncture as a means of giving relief to an inflamed testicle. The plan had served him well in about 500 cases. The relief obtained was immediate and permanent, and he believed arose from division to a small extent of the tense and unyielding tunica albuginea. The small amount of blood lost had nothing to do with it.—*Ibid.*

